# The POSE study - panic control treatment versus panic-focused psychodynamic psychotherapy under randomized and self-selection conditions: study protocol for a randomized controlled trial

**DOI:** 10.1186/s13063-015-0656-7

**Published:** 2015-03-31

**Authors:** Rolf Sandell, Martin Svensson, Thomas Nilsson, Håkan Johansson, Gardar Viborg, Sean Perrin

**Affiliations:** Department of Psychology, Lund University, Box 213, Lund, 221 00 Sweden; Institute of Psychiatry, Psychology and Neurosciences, Box PO77, King’s College London, 16 DeCrespigny Park, London, SE5 8AF UK

**Keywords:** Randomized preference trial, Panic disorder, CBT, Psychodynamic therapy, Health economics

## Abstract

**Background:**

Panic disorder with or without agoraphobia is a commonly occurring disorder affecting 2 to 3% of the population in Sweden. Untreated, panic disorder is a chronic condition that significantly increases the risk for psychiatric comorbidity, morbidity and mortality, employment difficulties, and healthcare utilization. Cognitive behavioral approaches are the recommended first-line treatment for panic disorder; however, many patients in routine care receive another evidence-based psychotherapy, including psychodynamic therapy. Allowing patients to choose among evidence-based approaches to panic disorder may improve outcomes and reduce overall health costs. Trials comparing the ‘gold standard’ treatment for panic disorder to other evidence-based psychotherapies are needed, and also trials that can separate patient preferences for treatment from randomization effects on outcome, disability and healthcare utilization in the longer term.

**Methods/Design:**

A phase 2/3 doubly-randomized controlled trial carried out in routine care with 216 adults (aged 18 to 70 years) with a primary diagnosis of DSM-IV Panic Disorder (with or without Agoraphobia). Within each clinic, patients are randomized to self-selection, random assignment of treatment, or wait-list. Patients choose or are randomly assigned to either Panic Control Treatment or Panic-Focused Psychodynamic Psychotherapy. Primary outcomes are changes in panic symptom severity, occupational status, and sickness-related absences from work at post-treatment and 6, 12 and 24 months post-treatment. Secondary outcomes include changes in agoraphobic avoidance, psychiatric comorbidity, disability, and healthcare utilization. The study also employs elements of an effectiveness trial as therapist and service-related effects on outcome will be estimated. Putative change mechanisms for the two treatments are also assessed.

**Discussion:**

Cognitive behavioral and psychodynamic therapies are both evidence-based approaches that are routinely offered to panic disordered patients in Sweden. However, little is known about the relative effectiveness of these two approaches for panic/agoraphobia, work-related disability and healthcare utilization over the longer term. The current trial (POSE) also addresses the important but understudied issue of whether patient preference for a particular psychotherapeutic approach moderates outcome.

**Trial registration:**

ClinicalTrials.gov NCT01606592 (registered 19 March 2012).

## Background

Panic Disorder (PD) without Agoraphobia as defined in the 4th edition of the *Diagnostic and Statistical Manual for Mental Disorders* (DSM-IV) (American Psychological Association, 2000) involves recurrent, unexpected panic attacks with at least one attack followed by persistent concerns or worries about additional attacks, and/or significant changes in behavior related to the attacks. PD with Agoraphobia involves the additional requirement that the individual avoids or endures with marked distress (often necessitating a companion to accompany them) places or situations from which it might be difficult (or embarrassing) to escape and/or to receive help in the event of a panic attack [1]. In DSM-V, the two disorders are de-linked so that individuals with both PD and Agoraphobia receive two diagnoses [2]. As this study commenced prior to the publication of DSM-V, we used the DSM-IV criteria with treatment focused on patients with a primary diagnosis of PD - with or without Agoraphobia.

In accordance with prevalence studies carried out elsewhere [3,4], the 12-month prevalence of PD in Sweden is 2 to 3% and one of the most frequent causes of illness among Swedes aged 15 to 44 years [5]. When untreated, PD is a chronic and debilitating condition associated with an increased risk of psychiatric comorbidity, health complaints, low quality of life, diminished capacity for work, morbidity and mortality, a heavy burden on the family, and significantly increased healthcare utilization [5-10]. The presence of PD, especially with agoraphobia, is recognized as a major obstacle to Swedish adults returning to work after a period of sick leave and to young people attempting to enter the labor force for the first time (50% of whom with PD fail to do so) [5]. When last estimated (1996), the total cost of PD in Sweden was estimated at 1.7 billion euros per annum [5]. Effective treatment of PD has been shown to reduce the cost of care by as much as 94% [11].

Cognitive behavioral therapy (CBT) and pharmacotherapy are the recommended treatments for adults with PD in many countries [12-14] and the same is true in Sweden [5]. Numerous meta-analyses have been carried out and overall they find that both treatments, either alone or in combination, yield large effect sizes when compared with no/minimal treatment and medium effect sizes relative to psychological/drug placebo in the acute phases of treatment [15-17]. Effect sizes beyond 12 to 24 months are generally more modest than at post-treatment or 6 month follow-up, particularly for (mono) pharmacotherapy, with little evidence of further symptomatic improvement during the follow-up period [14-18]. The use of pharmacotherapy alone is also associated with a significantly increased risk of attrition during treatment and relapse when treatment is discontinued [15,19]. Thus, there is an emerging consensus that CBT should be offered first to PD patients and to those discontinuing pharmacotherapy [5,14,15,17]. Whether another evidence-based psychotherapy could also be offered remains unclear, as there have been extremely few trials involving PD patients and comparisons between CBT and an active psychological treatment [20].

The longer-term efficacy of CBT for PD also remains unclear [17,21]. Across randomized controlled trials (RCTs) the median length of follow-up is 6 months with few trials reporting findings past 12 months. Also, few trials involve designs that permit continued comparison between treatments or between treatment and control groups during the follow-up period. Conclusions about longer-term efficacy are further confounded by significant attrition and treatment-seeking during the follow-up period.

Durham *et al*. [21] carried out a 2- to 14-year follow-up of participants in 8 RCTs of CBT for anxiety, 4 of which focused on PD and the others on Generalized Anxiety and Post-Traumatic Stress Disorders (PTSD). Of those initially treated with CBT for PD, only 40% achieved sustained recovery (defined as the absence of any anxiety disorder) without the benefit of additional treatment (any) during the follow-up period. Even with additional treatment, only 54% of those initially treated with CBT for PD achieved long-term recovery [21]. Across all of the RCTs, ‘recovered’ patients had significantly worse scores on all subscales of the Brief Symptom Inventory, with most suffering from mild symptoms that were slightly disabling and 20% falling just below threshold for diagnosis. More favorable findings can be found in a recent multi-center RCT by Gloster *et al*. [22] comparing 2 forms of CBT for PD as 74% of patients who were responders at post-treatment retained this status at 24 months. However, 37.5% of all CBT treated patients sought further treatment between 6 and 24 months and this was unrelated to anxiety severity at 6 months. Further favorable findings are available from an earlier uncontrolled study of PD patients treated with CBT wherein 82% of initial treatment responders remained in remission at 5 or more years post-treatment and 62% at 10 years or more [23]; however, the patients were less ill to begin with.

It is important to note that most RCTs involving PD patients have focused primarily on changes in symptoms (panic, agoraphobia, and depression) and general indices (often clinician-rated) of overall impairment. Less is known about the impact of evidence-based approaches on the other aspects of disability that usually accompany PD such as absences from work due to sickness, difficulties returning to work and increased healthcare utilization [24]. Reducing symptoms *per se* does not necessarily improve these aspects of functioning [24-26]. There is some evidence that short-term therapies are less effective than longer therapies on improving return to work after sick leave in depressed and anxious patients [24,27]. However, the general move in health services overall is towards briefer, not longer, psychological treatments.

What about attrition in CBT and pharmacotherapy? PD patients fear somatic symptoms and often the medical side effects that can accompany pharmacotherapy, and these fears may represent a major reason why PD patients often refuse medication when first offered (pre-treatment attrition) or why as many as one third prematurely discontinue pharmacotherapy once begun [28,29]. Limited data is available about pre-treatment attrition in relation to CBT. In a study of 731 patients (22% with PD) presenting to a specialist anxiety service, all of whom were offered CBT outside of a clinical trial, the authors found that 30% dropped out before commencing CBT with 12.6% actively refusing CBT [30]. Although drop-out from CBT once begun was relatively low in the previous study (10.7%), a meta-analysis of RCTs by Haby *et al*. involving patients with either primary PD, Generalized Anxiety Disorder, or depression reported an average drop-out rate of 17% for PD patients treated with CBT (range = 0 to 54%) [18].

The dominance of CBT in the treatment of anxiety over the past two decades has meant that relatively few RCTs have been carried out comparing this approach to another active psychological treatment [31,32]. The majority of comparative psychotherapy trials for patients with PD have involved two forms of CBT (for example, cognitive versus behavioral, with/without therapist-guided exposure, self-help, Internet-based, and so on). Psychodynamic therapy (PDT) is an evidence-based treatment for a range of conditions involving a significant anxiety component [32,33]. While much fewer RCTs of PDT exist than for CBT, meta-analytic reviews have been carried out to estimate the effectiveness of PDT for a range of problems including anxiety [34-38]. In the 2 most recent [35,36], PDT was observed to be no less effective for anxiety than other active treatments in the short or long term, had similar drop-out rates (17%) as reported for CBT, and yielded controlled effect sizes relative to no/minimal treatment or treatment as usual in the medium range (*g* = 0.63). These effects were persistent at follow-ups of 6 to 12 months; however, the limited number of trials (focused primarily on social anxiety) and the significant heterogeneity between trials limits the conclusions that can be drawn [36]. Further comparative trials involving PDT and CBT are needed [13].

Milrod *et al*. [39] have developed a brief form of PDT titled *Panic-Focused Psychodynamic Psychotherapy* (PFPP). In an open trial of PFPP with 21 panic disordered patients (most with comorbid agoraphobia), 16 (76%) experienced a remission of their panic/agoraphobic symptoms at post-treatment with gains maintained at 6 months [40,41]. In a subsequent RCT involving 49 PD patients, those receiving PFPP were significantly more likely to have their panic symptoms remit at termination than patients receiving applied relaxation training (73% versus 39%) with PFPP gains (and differences between the groups) maintained at 12 months [42]. PFPP was well-tolerated with only 7% withdrawing prematurely as compared to 34% in the applied relaxation condition. More recently, Beutel *et al*. carried out a randomized controlled trial of PFPP and CBT (plus exposure) delivered in routine care to 54 patients with PD (the majority with agoraphobia) [43]. Remission rates at post-treatment were 44% for PFPP rising to 50% at 12-month follow-up compared to a 66% remission at post-treatment for CBT decreasing to 56% at follow-up. The differences between the two treatments were non-significant.

While further comparative trials between PFPP and CBT for PD are clearly needed, Brewin and Bradley [44] have argued that RCTs comparing two or more treatments may give misleading results if patients’ preferences for the offered treatment alternatives are not controlled for. Thus, to the extent that patients’ preferences reflect their suitability for a specific type of psychotherapy, the one that is the most suitable for the majority of the patient population will appear superior, even if the treatments are equally effective. In a qualitative follow-up study of patients who had received CBT and PDT, patients who had unsuccessful treatment felt they had received a form of therapy that was less suitable to their individual needs and disposition and had well-developed ideas about how a more effective treatment should have been carried out [45]. A series of studies have shown that most people given adequate information will voice definite ideas about the kind of treatment that might be most suitable to their needs: so-called ‘helpfulness beliefs’ [46-48]. The resulting preferences are significantly moderated by the patient’s previous experiences with psychotherapy or psychiatry in general and generate a distribution of what may be a proxy for the ‘suitability differential’ between different forms of psychotherapy [48].

So, in a trial comparing two or more treatments where patients are allowed to select their preferred evidence-based treatment, one might reasonably predict little or no difference between the treatment groups. Also, if one assumes that outcome is at least partly moderated by the quality of the treatment alliance, one might reasonably expect stronger effects generally for treatments that were patient-selected than randomly assigned. It is possible to separate the effects of patient preference from the effects of randomization (that is treatment) by using a hybrid, double-randomization design [49]. In such a trial patients are randomized to self-selection or random assignment of treatment conditions.

While patient preferences have drawn considerable attention as a potential moderator of treatment outcome [46-48,50,51], no such hybrid randomized controlled study (comparing treatment and preference effects) has yet been published for PD patients. A recent study by Watzke *et al*. [52] compared systematic treatment selection to randomized assignment of treatment, either non-protocol CBT or PDT, on outcomes for a wide range of disorders (including anxiety) in 291 inpatients. In the systematic treatment selection condition, the inpatient team arrived at a consensus decision about the most appropriate treatment based on diagnosis, psychometric assessment, capacity for self-reflection, and patient goals. The authors found no overall differences in outcome between patients in the randomized assignment and systematic treatment selection conditions. Patients who were systematically assigned to PDT had better long-term outcomes than patients who were randomly assigned to this same treatment but no such effects were found for CBT.

The role of patient preference for a particular evidence-based approach is better illustrated in a recent hybrid RCT preference trial by Le *et al*. [53]. The investigators randomized 218 patients with a primary diagnosis of PTSD to patient-preference or randomly-assigned treatment conditions involving either prolonged exposure therapy (a form of CBT) or sertraline. Overall, giving patients a choice over treatment yielded significantly lower health costs and more quality-adjusted life years (QALYs) than random assignment to treatment conditions. Interestingly, prolonged exposure therapy was less costly and yielded more QALYs relative to sertraline when the treatment was assigned rather than chosen.

CBT and pharmacotherapy are currently the ‘gold standard’ treatments for PD. Pharmacotherapy will remain an important treatment option for many patients but pre- and post-treatment attrition for pharmacotherapy remains a significant concern. CBT is clearly an effective approach for PD but not for every patient and longer-term gains remain somewhat unclear. Also, the state of the literature is such that those advocating for the increased use of CBT in routine care for patients with PD will struggle to point to data showing its incremental effectiveness over other evidence-based psychotherapies that are also routinely provided in mental health services. Increasingly, both healthcare service providers and treatment guidelines have recommended that patients be encouraged and supported in choosing between various treatment options and being more active in the way treatment is delivered. It is possible that allowing patients to choose among evidence-based approaches may improve outcomes and reduce the overall burden and cost of illness. Further trials are needed involving comparisons between CBT and other evidence-based psychological approaches to help patients make better, data-informed choices about which treatment is likely to work best for them in the short and longer term.

### Study aims and objectives

The primary aim of the project is to investigate the extent to which patient’s preferences for treatment influences a broad range of primary and secondary outcomes: panic symptom severity, agoraphobic avoidance, occupational status, sickness-related absences, subjective working ability, psychiatric comorbidity, panic and depression-related disability, and healthcare/medication utilization. A secondary aim is to compare outcomes for PCT, which is the form of CBT with the largest evidence base for PD, to PFPP, both of which are already delivered in routine care facilities in Sweden. A tertiary aim is to evaluate in a multivariate fashion, interactions between patient preference and other potential moderators of outcome (for example, gender, age, symptom severity, Axis I and II comorbidity, medication use, strength of the therapeutic alliance, and therapist effects). Finally we aim to evaluate putative mechanisms of change associated with each of the evaluated treatments (for example, cognitive and behavioral change, reflective capacity).

## Methods/Design

### Study design

PCT and PFPP will be compared under two conditions, one in which patients are randomized to either form of therapy, and one in which they are asked to choose between them after having duly informed themselves about the two alternatives. The design is in keeping with recommendations for preference trials described by Long *et al*. [49].

### Setting

The study is carried out in ten outpatient mental health clinics located in three regions of Southern Sweden: Skåne (including Lund, Malmö, Eslöv, Arlöv and Trelleborg), Halland (including Halmstad, Falkenberg and Varberg), Jönköping (including Värnamo) and Dalecarlia (including Dalarna). These clinics are state-funded and responsible for the care and treatment of all individuals within their catchment area. The clinics receive referrals from the GP (family doctor), social services, employers, and directly from patients. In some cities some patients may be offered a brief psychological treatment in what are termed ‘first-line’ services (usually located in the GP clinics). However, and in the majority of cases, the clinics participating in this study are the first-line service for patients with mental health problems including panic and other anxiety disorders, and particularly those with significant psychiatric comorbidity or impairment in functioning. PCT and PFPP are delivered by therapists employed in the same clinics where the patient would ordinarily be treated.

### Participant inclusion criteria

We aim to maximize the external validity of the study to the extent possible by making the inclusion criteria as least restrictive as possible. Inclusion criteria are the following:

- Aged between 18 and 70 years
- Current, principle diagnosis of DSM-IV PD with/without Agoraphobia and more specifically:
- Have experienced at least 1 panic attack per week during the 3 weeks preceding the start of treatment; or
- If PD with Agoraphobia is the principal diagnosis and the patients actively avoids situations that cause them panic, then the patient must:
- Score ≥ 5 on an apprehension question about having a panic attack; or
- Score ≥ 4 on the Avoidance-Alone Subscale of the Mobility Inventory for Agoraphobia; or
- Meet DSM-IV PD criteria and *not* be more appropriately diagnosed as Agoraphobia without a history of PD;
- Not currently engaged in on-going psychotherapy treatments and willing to refrain from starting non-study treatments during the treatment phase of the trial (discussed further below under screening and baseline assessment)
- Medications, if used, must be held constant throughout the treatment phase of the study, and patients will only be accepted if they have been on a stable dose of standard anti-panic medication for at least 1 month and continue to meet criteria for a current diagnosis of PD with or without Agoraphobia; and
- Ability to complete the active treatment phase (not including follow-ups) within 16 weeks

### Participant exclusion criteria

- Current substance abuse/dependence. Patients with a prior history of substance abuse/dependence must have been in remission for this condition for at least 12 months prior to entering trial treatment; or
- Current psychosis, delusions, or mania; or
- Acutely suicidal; or
- A history and current clinical presentation of at least one clinically-significant medical condition (for example, brain damage, degenerative neurological condition) sufficient to cause cognitive or physical impairments that might prevent full participation in treatments; or
- Active involvement in a legal dispute related to their mental health

### Recruitment procedure

This trial is carried out with significant support from regional health authorities and thus the primary means of recruitment is through state-funded, outpatient mental health clinics. Information about the study is posted in waiting rooms and all clinic staff are made aware of the study and asked to inform new referrals and existing patients who suffer from problems related to anxiety and panic specifically about the trial. Information about the trial is also available through a Project POSE website. Individuals with panic-related problems who visit the website are invited to contact the researchers for further information about the trial and an assessment of trial eligibility. Recruitment levels at the ten different sites are monitored on a monthly basis and problems addressed at the appropriate organizational level. Protocols governing inclusion have been developed and disseminated to facilitate a strict and coherent plan of action across sites, treatments conditions, therapists and site managers should problems arise.

### Screening and baseline assessment

When a clinic identifies a potential participant, or when a participant directly contacts a member of the research team, an initial phone screening is carried out and the patient is informed about the trial, and inclusion and exclusion criteria are discussed. Patients who are currently receiving psychotherapy for PD or any other condition are strongly advised to complete their current course of treatment and then re-contact the team if they are still interested in participation. (At the time of writing, 138 patients have been enrolled in the trial. Of these, none were receiving ongoing psychotherapy for any condition at the time of screening. Only two patients who were receiving psychotherapy for PD expressed an interest in the trial and both were asked to complete their treatment before re-contacting the team and were not enrolled). Patients who are currently receiving medication for anxiety or any other psychiatric condition are advised to meet with their psychiatrist and to bring a copy of the trial information form to discuss participation and the trial criteria regarding medication before agreeing to further assessment for the trial.

Provided the patient has problems primarily of an anxious nature, wants treatment and does not meet any of the exclusion criteria, a face-to-face assessment is scheduled. Before this assessment the patient is provided with further information about the trial to read and a range of questionnaires to complete (described below). During the face-to-face assessment the patient undergoes a structured diagnostic interview with a member of the research team to establish that DSM-IV PD with or without Agoraphobia is the current and principal diagnosis, the Panic Disorder Severity Scale is administered, and lastly a structured diagnostic interview is carried out to assess DSM-IV Axis II Personality Disorders. If the patient has PD with or without Agoraphobia and no exclusion criteria are met, the patient gives written consent to participate and is then randomized. Patients who are excluded at any time during the baseline assessments are encouraged to contact their local GP and/or mental health clinic, and where requested/necessary a formal letter of referral is prepared by the trial assessor.

### Inclusion measure

In keeping with the recommendations for standardized assessment of panic disordered patients described by Shear and Maser [54] and followed in most RCTs with panic disordered patients, inclusion is based on diagnostic status as assessed by the Structured Clinical Interview for DSM-IV (SCID-I) [55].

### Primary outcome measures

- Panic Disorder Severity Scale [56]
- Panic Disorder Severity Scale-Self Report [57]
- Occupational status
- Patient report of the total of number of absences from work due to sickness (and compensation for the same as part of the secondary, healthcare utilization outcome)

The above outcome measures are all assessed at baseline, post-treatment and 6-, 12- and 24-month follow-ups. The total of number of absences from work due to sickness at baseline is measured as including the 3 months prior to commencing treatment.

### Secondary outcome measures

- Psychiatric comorbidity as assessed by the Structured Clinical Interview for DSM-IV Axis I diagnoses [55] and Structured Clinical Interview for DSM-IV Axis II diagnoses [58]
- Mobility Inventory for Agoraphobia [59]
- Montgomery Åsberg Depression Rating Scale [60]
- Sheehan Disability Scale [61]
- Clinical Outcomes in Routine Evaluation [62]
- Work Ability Index [63]
- Number of absences from work due to sickness before, during and after treatment as recorded in the Longitudinal Integration Database for Health Insurance and Social Studies (LISA) that is maintained by the Swedish Central Statistical Office
- Patient report of healthcare utilization (for example, number of medical contacts, emergency room visits, prescription medication use)
- Use of psychotropic medication as recorded by the Swedish National Board of Health and Welfare register of consumption of psychotropic drugs; and
- Estimation of the total expected costs of each treatment alternative against the total expected benefits

### Randomization and allocation concealment

Randomization is done sequentially as patients are included in study. At the level of the clinic, there must be at least one PFPP and one PCT therapist working in the trial. The randomization procedure was designed to ensure equal numbers of patients in each condition at clinic level.

Because of the length of the follow-up (24 months) and the heavy assessment burden for patients throughout the trial, there is an increased risk of attrition during the follow-up period. To help minimize this risk, an effort is made to establish a good working relationship between a single trial assessor (who is not involved in delivering treatment) and each patient and to have the same assessor carry out the baseline, post-treatment and 6-, 12- and 24-month follow-up assessments. Because the baseline assessor reveals the randomization results to the patient, the post-treatment and follow-up interviews are not carried out blind to treatment condition. However all post-treatment and follow-up interviews are recorded and these interviews will be evaluated by independent raters who are blinded to treatment condition and these ratings will be reported.

### Treatment protocol

PCT is a manualized, individual cognitive behavioral treatment for adults with PD (with or without agoraphobia) [64]. Patients are helped to become panic-free by learning to anticipate and respond to situations that trigger their panic attacks and to manage the physical symptoms of panic using techniques such as controlled breathing. PCT ordinarily consists of 12 weekly therapy sessions. In the current trial, PCT involves 12 to 14 sessions completed within 10 to 16 weeks; week 1 includes 2 sessions and subsequent weeks 1 session each. Between 2 and 5 sessions will include therapist-assisted exposure. Each session is 60 minutes in duration, extended to 90 to 120 minutes for those including therapist-assisted exposure. Total treatment duration will be 780 to 1,140 minutes.

Session 1 of PCT involves psycho-education about the nature of PD and agoraphobia. In Session 2 the patient learns the importance of (and how to) recording their daily levels of anxiety/panic so that they can monitor progress alongside their therapist. Sessions 3 to 12 involve cognitive restructuring of anxiety-related cognitions and structured, therapist-guided exposure to situational/somatic cues that may be associated with panic attacks and agoraphobic avoidance. A single session teaches clients how to slow their breathing rate to counter the effects of hyperventilation.

PFPP is a manualized, individual psychodynamic treatment for adults with PD (with or without agoraphobia) [38]. Treatment is ordinarily 19 to 24 sessions in length with 2 sessions per week, 45 minutes each. PFPP delivered in the current trial comprises 19 to 24 sessions completed within 10 to 16 weeks; in principle 2 sessions per week, 45 minutes each. Total treatment duration will be 855 to 1,080 minutes.

PFPP proceeds in three phases. In Phase I the therapist works to identify the specific content and meanings of the panic episodes and to help the patient examine the stressors and feelings surrounding the onset and persistence of panic. Specific vulnerabilities to the development of panic from the patient’s past are explored (for example, representations of parents, traumatic experiences, difficulty expressing and managing angry feelings). The patient is aided in bringing forth fantasies and feelings that may have been unconscious or difficult to tolerate, such as vengeful wishes or abandonment fears, and to identify intra-psychic conflicts surrounding anger, separation, and sexuality. The goal of this phase is reduction in panic symptoms.

Phase II seeks to address the dynamics that maintain vulnerability to panic and the persistence of panic symptoms (for example, difficulties with anger recognition and management, separation, and fears of loss or abandonment). These dynamics are addressed through discussion of the patient’s feelings/fantasies about past/present relationships and in the transference relationship with the therapist. Improved understanding of these conflicts helps to prevent the development of a vicious cycle of PD recurrence.

In Phase III the therapist and patient work with the patient’s conflicts with anger and separation as they emerge in the context of termination. Increased assertiveness and the capacity to communicate about conflicts in relationships should improve quality of life and reduces panic vulnerability. Thus the patient is helped to experience and articulate their feelings about loss and anger directly with the therapist to facilitate better management of these feelings and the capacity to avert the development of more severe panic states.

### Treatment integrity

Treatment integrity refers to the degree to which an intervention is delivered as intended. Therapists in the trial provide only one form of treatment during the study and the vast majority will provide only CBT or only PDT as part of their usual practice. Treatment integrity encompasses three aspects: a) therapist treatment adherence (the degree to which the therapist utilizes prescribed procedures and avoids proscribed procedures); b) therapist competence (the level of the therapist’s skill and judgment); and c) treatment differentiation (whether competing treatments differ from each other along critical dimensions).

All three aspects of treatment integrity are addressed throughout the trial in keeping with the standards for the field proposed by Perepletchikova *et al*. [65]. The first two aspects are addressed firstly through training and supervision of trial therapists in PCT and PFPP prior to their participation in the study. Prior to commencement of patient recruitment therapists and supervisors receive workshops in PCT and PFPP and additional workshops are arranged each year of the trial. Therapists must also treat as per-protocol (videotaping sessions) a ‘training’ case under supervision from a trial-trained supervisor. Videotapes from this therapy are evaluated by another experienced supervisor of PCT or PFPP for treatment adherence. During the trial therapists continue to videotape treatment sessions and to receive supervisions from a trial-trained supervisor. The supervisor rates therapist adherence during supervision and provides feedback to the therapist about the same. Adherence is also controlled for each patient therapy by three ratings (beginning, middle and end of therapy) made by independent raters with feedback from these ratings given to trial supervisors to use with therapists. A minimum of three site visits per annum from the research team are scheduled to ensure (improve) site protocol adherence.

### Project implementation plan

Point prevalence of PD in the Swedish general population is 2 to 3% [5], which means that over 40,000 individuals currently have PD in the regions of Halland, Skåne and Dalecarlia. Given an inclusion period of 6 years (2011 to 2016) about 40 patients have to be recruited each year. Trial therapists are expected to treat about three patients each per year. The study is carried out at 10 sites in the counties of Skåne (Lund, Malmö, Eslöv, Arlöv and Trelleborg), Halland (Halmstad, Falkenberg and Varberg), Jönköping (Värnamo) and Dalecarlia. All participating therapists work in primary healthcare centers or psychiatric outpatient clinics. Currently, 35 therapists are active in the project. All clinics have signed written agreements to supply the project with patients during the inclusion phase. Intake and treatment of patients began in 2011. This phase is scheduled to last 5 years until 2016. Follow-up studies will be completed in 2018.

### Power/sample size calculations

For the planned and relatively straightforward comparisons between the 2 active treatments (PCT and PFPP) and wait-list, 25 patients per group would be needed in order to ensure an effect size (Cohen’s *d*) of 0.8 at alpha = 0.05 and power = 0.80. Our assumption of an effect size difference versus wait-list of *d* = 0.8 is conservative relative to the average controlled effect size (*d* = 1.02, 95% CI = 0.86 to 1.18) reported in a recent meta-analysis of 61 RCTs comparing psychological treatments for PD to no treatment or psychological/drug placebo [17]. For the planned and straightforward comparison between the randomized and self-selection conditions, we have assumed that the difference between the two conditions will be in the order of *d* = 0.4. Again our assumption is conservative relative to the average effect size obtained for self-selection versus randomization in preference trials involving treatments for anxiety (*d* = 0.49, 95% CI = 0.19 to 0.79), albeit based on a much smaller number of RCTs [66]. For the more complex comparisons involving the hypothesized interaction between assignment type (randomization or self-selection) and treatment type (PCT or PFPP), power calculations have been performed based on the PINT (Power IN Two-level designs, v. 2.12, September 2007) [67,68]. In the absence of *a priori* information about the size of the interaction effect between assignment and these 2 specific treatments, we assume the interaction effect size will be comparable in size to the main effect size for assignment (that is *d* = 0.40). At alpha = 0.05, power = 0.80 and *d* = 0.4, a total of 200 patients is required. Assuming a 15% drop-out rate, a total of 212 patients is required.

After consideration of issues pertaining to practicality of randomization, the target recruitment is N = 216. This was based on the following tentative assumptions, among others: Assignment (randomized versus self-selected) is a fixed effect on Level 1 with M = 0 and SD = 1; the Pre-treatment measure is a random and fixed effect on Level 1; Treatment (PCT versus PFPP) is a Level 2 predictor with M = 0.0625 (given a 55:45 distribution in the self-selection group) and SD = 1; residual variance in post-treatment = 0.70; and explained variance on outcome across therapists = 0.05.

**Figure 1:**
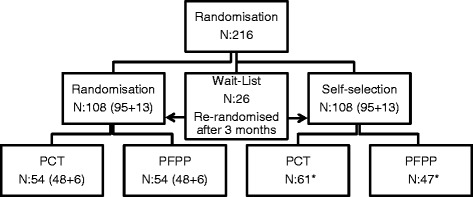
**Consolidated Standards of Reporting Trials (CONSORT) flow chart.** *Number of participants choosing PCT and PFPP in Self-selection condition is hypothetical and based on available studies.

The 216 patients will be randomly allocated to the randomized condition, the self-selection condition or to the waiting-list in the following shares: 108: 108: 26 (see Figure 1 for Consolidated Standards of Reporting Trials (CONSORT) flow chart). After 3 months patients on the waiting-list will be re-randomized to randomization or to self-selection. The self-selection condition allows the patient to choose either PFPP or PCT. The actual choice is made after reading a detailed and well-balanced presentation of the two alternative therapies. These presentations have been developed and tested in an unpublished study by Hultman.

### Ethical considerations

Ethical approval was obtained from the Regional Ethical Review Board in Lund (Ref: DNR-2010/88). Ethical and professional guidelines will be followed at all times, in line with Good Clinical Practice guidelines, and the laws governing patient care and data protection in Sweden. Informed consent is obtained from every participant in the trial. Any potential adverse events are discussed with the treating therapist, their clinical supervisor (and employer as necessary), and the research team (including the relevant university representative), and any identified adverse events are recorded. Adverse events are reported to the trial funder and will be reported as part of the trial findings.

## Discussion

The ability of patients to choose freely among treatments in matters of health is stipulated by law in Sweden but choice is often sacrificed in the name of cost-effectiveness. However, policy-makers would do well not to limit patient choice to the most cost-effective treatment based on often limited evidence, when an alternative evidence-based approach that is not more expensive than the most cost-effective therapy is available, but is as yet understudied. Allowing patients to choose among evidence-based approaches will increase patient involvement and may result in improved patient safety and patient satisfaction. Likewise, providing patients access to their preferred (evidence-based) treatment is likely to increase compliance rates, thereby improving outcomes and reducing the overall cost and burden of illness [69].

Currently, CBT is the indicated first-line treatment for adult patients with PD in Sweden but healthcare authorities recognize that other evidence-based approaches that are understudied may also be effective [5]. Swedish mental health services benefit from the presence in routine care of psychotherapists with post-graduate (certified) training in a range of evidence-based approaches, primarily CBT, PDT and family therapy. However, comparative trials of these various approaches to PD have not been carried out in Sweden. The current trial will be the first randomized controlled trial in Sweden to compare CBT (PCT) to brief psychodynamic therapy (PFPP) delivered in routine care to patients with PD. The trial is also important in the emphasis placed on measuring the impact of treatment on work-related disability and healthcare utilization, both of which are significant costs associated with PD in Sweden and elsewhere.

This project has the unique ambition to separate the effects of patients’ preferences for treatment from the effect of the different evidence-based psychotherapies under study. If a certain form of psychotherapy proves to be significantly more popular in the self-selection group, this is an interesting research finding in and of itself, which may ultimately have implications for how healthcare resources are allocated in Sweden and for the design of future comparative trials. Should PFPP prove to be as effective as the established ‘gold-standard’ treatment for PD (CBT), the current trial will significantly add to the literature and the evidence base in favor of offering PD patients further choices among evidence-based treatments.

## Trial status

Recruitment began in November 2011 and is ongoing. Slightly more than half of the target sample has been recruited (N = 138).

## Abbreviations

CBT: cognitive behavioral therapy
CONSORT: Consolidated Standards of Reporting Trials
DSM: Diagnostic and Statistical Manual for Mental Disorders
LISA: Longitudinal Integration Database for Health Insurance and Social Studies
PCT: panic control treatment
PD: Panic Disorder
PDSS: Panic Disorder Severity Scale
PDT: psychodynamic psychotherapy
PFPP: panic-focused psychodynamic psychotherapy
PINT: Power IN Two-level
PTSD: Post-Traumatic Stress Disorder
QALYs: quality-adjusted life years
RCT: randomized controlled trial
SCID-1: Structured Clinical Interview for DSM-IV

## References

[1] American Psychiatric Association (2005). Diagnostic and statistical manual of mental disorders.

[2] American Psychiatric Association (2013). Diagnostic and statistical manual of mental disorders.

[3] Goodwin RD, Faravelli C, Rosi S, Cosci F, Truglia E, De Graaf R (2005). The epidemiology of panic disorder and agoraphobia in Europe. Eur Neuropsychopharmacol.

[4] Kessler RC, Chiu WT, Jin R, Ruscio AM, Shear K, Walters EE (2006). The epidemiology of panic attacks, panic disorder, and agoraphobia in the National Comorbidity Survey Replication. Arch Gen Psychiatry.

[5] Statens Beredning för medicinsk utvärdering (SBU). Behandling av ångestsyndrom. Stockholm: SBU-rapport number 171; 2005.

[6] Batelaan N, Smit F, De Graaf R, Van Balkom A, Vollebergh W, Beekman A (2007). Economic costs of full-blown and subthreshold panic disorder. J Affect Disord.

[7] Batelaan NM, De Graaf R, Spijker J, Smit JH, van Balkom AJ, Vollebergh WA (2010). The course of panic attacks in individuals with panic disorder and subthreshold panic disorder: a population-based study. J Affect Disord.

[8] De Graaf R, Tuithof M, Van Dorsselaer S, Ten Have M (2012). Comparing the effects on work performance of mental and physical disorders. Soc Psychiatry Psychiatr Epidemiol.

[9] Nepon J, Belik SL, Bolton J, Sareen J (2010). The relationship between anxiety disorders and suicide attempts: findings from the National Epidemiologic Survey on Alcohol and Related Conditions. Depress Anxiety.

[10] Sherbourne CD, Sullivan G, Craske MG, Roy-Byrne P, Golinelli D, Rose RD (2010). Functioning and disability levels in primary care out-patients with one or more anxiety disorders. Psychol Med.

[11] Salvador-Carulla L, Segui J, Fernandez-Cano P (1995). Costs and offset effect in panic disorders. Brit J Psychiatry.

[12] American Psychiatric Association (2009). Practice guideline for the treatment of patients with panic disorder.

[13] NICE. Generalised anxiety disorder and panic disorder (with or without agoraphobia) in adults: management in primary, secondary and community care. NICE Clinical Guideline 113. 2011. http://www.nice.org.uk/guidance/cg113. Accessed 11 March 2015.

[14] Katzman MA, Bleau P, Blier P, Chokka P, Kjernisted K, Van Ameringen M (2014). Canadian clinical practice guidelines for the management of anxiety, posttraumatic stress and obsessive-compulsive disorders. BMC Psychiatry.

[15] Furukawa TA, Watanabe N, Churchill R (2007). Combined psychotherapy plus antidepressants for panic disorder with or without agoraphobia. Cochrane Database Syst Rev.

[16] Hofmann SG, Smits JA (2008). Cognitive-behavioral therapy for adult anxiety disorders: a meta-analysis of randomized placebo-controlled trials. J Clin Psychiatry.

[17] Sánchez-Meca J, Rosa-Alcázar AI, Marín-Martínez F, Gómez-Conesa A (2010). Psychological treatment of panic disorder with or without agoraphobia: a meta-analysis. Clin Psychol Rev.

[18] Haby MM, Donnelly M, Corry J, Vos T (2006). Cognitive behavioural therapy for depression, panic disorder and generalized anxiety disorder: a meta-regression of factors that may predict outcome. Aust N Z J Psychiatry.

[19] Farach FJ, Pruitt LD, Jun JJ, Jerud AB, Zoellner LA, Roy-Byrne PP (2012). Pharmacological treatment of anxiety disorders: current treatments and future directions. J Anxiety Disord.

[20] Arch JJ, Craske MG (2009). First-line treatment: a critical appraisal of cognitive behavioral therapy developments and alternatives. Psychiatr Clin North Am.

[21] Durham RC, Higgins C, Chambers JA, Swan JS, Dow MG (2012). Long-term outcome of eight clinical trials of CBT for anxiety disorders: symptom profile of sustained recovery and treatment-resistant groups. J Affect Disord.

[22] Gloster AT, Hauke C, Höfler M, Einsle F, Fydrich T, Hamm A (2013). Long-term stability of cognitive behavioral therapy effects for panic disorder with agoraphobia: a two-year follow-up study. Behav Res Ther.

[23] Fava GA, Rafanelli C, Grandi S, Conti S, Ruini C, Mangelli L (2001). Long-term outcome of panic disorder with agoraphobia treated by exposure. Psychol Med.

[24] Knekt P, Lindfors O, Laaksonen MA, Raitasalo R, Haaramo P, Järvikoski A (2008). Effectiveness of short-term and long-term psychotherapy on work ability and functional capacity - a randomized clinical trial on depressive and anxiety disorders. J Affect Disord.

[25] Elfering A (2006). Work-related outcome assessment instruments. Eur Spine J.

[26] Lazar A, Sandell R, Grant J (2007). Subjective health and ill health-related behaviour. Psychol Psychother.

[27] Mintz J, Mintz LI, Arruda MJ, Hwang SS (1992). Treatments of depression and the functional capacity to work. Arch Gen Psychiatry.

[28] Cowley DS, Ha EH, Roy-Byrne PP (1997). Determinants of pharmacologic treatment failure in panic disorder. J Clin Psychiatry.

[29] Marcus SM, Gorman J, Shear MK, Lewin D, Martinez J, Ray S (2007). A comparison of medication side effect reports by panic disorder patients with and without concomitant cognitive behavior therapy. Am J Psychiatry.

[30] Issakidis C, Andrews G (2004). Pretreatment attrition and dropout in an outpatient clinic for anxiety disorders. Acta Psychiatr Scand.

[31] Busch FN, Milrod BL (2010). The ongoing struggle for psychoanalytic research: some steps forward. Psychoanal Psychother.

[32] Shedler J (2010). The efficacy of psychodynamic psychotherapy. Am Psychol.

[33] Fonagy P, Roth A, Higgitt A (2005). The outcome of psychodynamic psychotherapy for psychological disorders. Clin Neurosci Res.

[34] Abbass AA, Hancock JT, Henderson J, Kisely S (2006). Short-term psychodynamic psychotherapies for common mental disorders. Cochrane Database Syst Rev.

[35] Abbass AA, Kisely SR, Town JM, Leichsenring F, Driessen E, De Maat S (2014). Short‐term psychodynamic psychotherapies for common mental disorders. Cochrane Database Syst Rev.

[36] Keefe JR, Mccarthy KS, Dinger U, Zilcha-mano S, Barber JP (2014). A meta-analytic review of psychodynamic therapies for anxiety disorders. Clin Psychol Rev.

[37] Leichsenring F, Rabung S, Leibing E (2004). The efficacy of short-term psychodynamic psychotherapy in specific psychiatric disorders: a meta-analysis. Arch Gen Psychiatry.

[38] Leichsenring F, Rabung S (2008). Effectiveness of long-term psychodynamic psychotherapy: a meta-analysis. J Am Med Assoc.

[39] Milrod BL, Busch FN, Cooper AM, Shapiro T (1997). Manual of panic-focused psychodynamic psychotherapy.

[40] Milrod B, Busch F, Leon AC, Shapiro T, Aronson A, Roiphe J (2000). Open trial of psychodynamic psychotherapy for panic disorder: a pilot study. Am J Psychiatry.

[41] Milrod B, Busch F, Leon AC, Aronson A, Roiphe J, Rudden M (2001). A pilot open trial of brief psychodynamic psychotherapy for panic disorder. J Psychother Pract Res.

[42] Milrod B, Leon AC, Busch F, Rudden M, Schwalberg M, Clarkin J (2007). A randomized controlled clinical trial of psychoanalytic psychotherapy for panic disorder. Am J Psychiatry.

[43] Beutel ME, Scheurich V, Knebel A, Michal M, Wiltink J, Graf-Morgenstern M (2013). Implementing panic-focused psychodynamic psychotherapy into clinical practice. Can J Psychiatry.

[44] Brewin CR, Bradley C (1989). Patient preferences and randomised controlled trials. Br Med J.

[45] Nilsson T, Svensson M, Sandell R, Clinton D (2007). Patients’ experiences of change in cognitive-behavioural therapy and psychodynamic therapy: a qualitative comparative study. Psychother Res.

[46] Bragesjö M, Clinton D, Sandell R (2004). The credibility of psychodynamic, cognitive and cognitive-behavioural psychotherapy in a randomly selected sample of the general public. Psychol Psychother.

[47] Frövenholt J, Bragesjö M, Clinton D, Sandell R (2007). How do experiences of psychiatric care affect the perceived credibility of different forms of psychotherapy?. Psychol Psychother.

[48] Sandell R, Clinton D, Frövenholt J, Bragesjö M (2011). Credibility clusters, preferences, and helpfulness beliefs for specific forms of psychotherapy. Psychol Psychother.

[49] Long Q, Little RJ, Lin X (2008). Causal inference in hybrid intervention trials involving treatment choice. J Am Stat Assoc.

[50] Arnkoff DB, Glass CR, Shapiro SJ, Norcross JC (2002). Expectations and preferences. Psychotherapy relationships that work.

[51] Swift JK, Callahan JL (2009). The impact of client treatment preferences on outcome: a meta-analysis. J Clin Psychol.

[52] Watzke B, Rüddel H, Jürgensen R, Koch U, Kriston L, Grothgar B (2010). Effectiveness of systematic treatment selection for psychodynamic and cognitive-behavioural therapy: randomised controlled trial in routine mental healthcare. Br J Psychiatry.

[53] Le QA, Doctor JN, Zoellner LA, Feeny NC (2014). Cost-effectiveness of prolonged exposure therapy versus pharmacotherapy and treatment choice in posttraumatic stress disorder (the Optimizing PTSD Treatment Trial): a doubly randomized preference trial. J Clin Psychiatry.

[54] Shear MK, Maser JD (1994). Standardized assessment for panic disorder research: a conference report. Arch Gen Psychiatry.

[55] First MB, Spitzer RL, Gibbon M, Williams JBW (1996). Structured clinical interview for DSM-IV Axis I.

[56] Shear MK, Brown TA, Barlow DH, Money R, Sholomskas DE, Woods SW (1997). Multicenter collaborative panic disorder severity scale. Am J Psychiatry.

[57] Houck PR, Spiegel DA, Shear MK, Rucci P (2002). Reliability of the self-report version of the panic disorder severity scale. Depress Anxiety.

[58] First MB, Spitzer RL, Gibbon M, Williams JBW, Benjamin LS (1997). Structured clinical interview for DSM-IV Axis II personality disorders (Version 2.0).

[59] Chambless DL, Caputo GC, Jasin SE, Gracely EJ, Williams C (1985). The Mobility Inventory for Agoraphobia. Behav Res Ther.

[60] Montgomery SA, Asberg M (1979). A new depression scale designed to be sensitive to change. Br J Psychiatry.

[61] Sheehan DV (1983). The anxiety disease.

[62] Evans C, Mellor-Clark J, Margison F, Barkham M, Audin K, Connell J (2007). CORE: clinical outcomes in routine evaluation. J Men Health.

[63] Ilmarinen J, Tuomi K, Klockars M (1997). Changes in the work ability of active employees over an 11-year period. Scan J Work Env Health.

[64] Barlow DH, Craske MG (1994). Mastery of your anxiety and panic.

[65] Perepletchikova F, Treat TA, Kazdin AE (2007). Treatment integrity in psychotherapy research: analysis of the studies and examination of the associated factors. J Consult Clin Psychol.

[66] Swift JK, Callahan JL, Vollmer BM (2011). Preferences. J Clin Psychol.

[67] Bosker RJ, Snijders TAB, Guldemond H (2003). PINT (Power IN Two-level designs): estimating standard errors of regression coefficients in hierarchical linear models for power calculations. User’s manual (Version 2.1).

[68] Snijders TAB, Bosker RJ (1998). Standard errors and sample sizes for two-level research. J Educ Stat.

[69] Brazier JE, Dixon S, Ratcliffe J (2009). The role of patient preferences in cost-effectiveness analysis: a conflict of values?. Pharmacoeconomics.

